# Dual-functional nitrogen-doped carbon dot/copper oxide nanocomposites for electrochemical sensing of ascorbic acid and antimicrobial applications

**DOI:** 10.1186/s13065-025-01639-3

**Published:** 2025-11-05

**Authors:** Zerfu Haile Robi, Dugasa Jabesa Nemera, Tekileab Engida Gebremichael, Guta Gonfa Muleta

**Affiliations:** 1 Department of Chemistry, Oda Bultum University, Oda Bultum, Chiro, Ethiopia; 2 Department of Chemistry, Jimma University, Jimma, Ethiopia; 3 Department of Chemistry, Gambella University, Gambella, Ethiopia

**Keywords:** Nitrogen-doped carbon dots, Copper oxide nanoparticles, Ascorbic acid detection, Electrochemical sensor, Antimicrobial activity

## Abstract

**Background:**

Antimicrobial resistance poses a growing threat to effective infection treatment, while imbalances in ascorbic acid (AA) levels are linked to various health issues. Nanotechnology offers innovative solutions, with carbon dot-based nanocomposites emerging as promising materials for both antimicrobial applications and sensitive detection of biomolecules. This study aimed to synthesize nitrogen-doped carbon dots (NCDs), copper oxide nanoparticles (CuO NPs), and their nanocomposites (CuO-NCDs), and to evaluate their electrochemical sensing of AA and antimicrobial activity.

**Methods:**

NCDs were prepared via a hydrothermal treatment of citric acid and urea, and CuO NPs by precipitation of copper nitrate. Characterization by Ultraviolet–Visible (UV–Vis) spectroscopy, Fourier Transform Infrared (FT-IR) spectroscopy, X-ray Diffraction (XRD), and Scanning Electron Microscopy (SEM) confirmed their optical properties, functional groups, crystallinity, and morphology.

**Results:**

The CuO-NCD nanocomposite exhibited enhanced optical absorption with a reduced energy band gap (2.6 eV) compared to individual components. Electrochemical tests revealed a detection limit of 2.56 µM for AA and increased electrode surface area, indicating improved sensitivity and selectivity. Antimicrobial assays showed significant activity against *Staphylococcus aureus*, with a 24 mm inhibition zone at 200 mg/mL after 24 h.

**Conclusions:**

These results demonstrate that CuO-NCD nanocomposites hold potential as dual-function materials for effective AA detection and combating microbial infections.

## Introduction

Antimicrobial resistance (AMR) has become a pressing global health issue due to the widespread misuse and overuse of antibiotics [1]. Multidrug-resistant (MDR) bacteria and fungi are increasingly difficult to treat and pose significant threats to human, animal, and environmental health [2]. This escalating crisis demands the development of novel antimicrobial strategies and sensitive detection systems to identify infections and reduce reliance on conventional antibiotics [3].

One area of growing interest is the application of nanotechnology to combat AMR and improve bioanalytical sensing [4]. Nanotechnology, the manipulation of materials at the nanoscale (1–100 nm), provides a versatile foundation for creating functional materials with unique chemical, optical, and electronic characteristics [5]. Due to their high surface-area-to-volume ratio, nanoparticles (NPs) exhibit greater reactivity and multiple functions in comparison to their larger counterparts [6]. In particular, nanocomposites (NCs) formed by integrating two or more nanomaterials, such as metal oxides with carbon-based structures, demonstrate synergistic effects that enhance their biological and electrochemical performance [2, 4].

Among these, carbon dots (CDs) have become a promising category of carbon-based nanomaterials because of their straightforward synthesis, low toxicity, biocompatibility, and excellent electron transfer capability [7]. Nitrogen-doped carbon dots (NCDs), prepared from nitrogen-rich precursors such as urea, exhibit improved conductivity and enriched surface functionality, which enhance their interaction with biological analytes [8]. When incorporated into metal oxide matrices like copper oxide (CuO), these NCDs can form hybrid nanocomposites with improved antimicrobial and electrochemical properties [9]. CuO is particularly attractive due to its redox activity, environmental friendliness, and capacity to produce reactive oxygen species (ROS), which contribute to microbial inhibition [10].

Ascorbic acid is vital for human health as a key antioxidant, involved in the synthesis of collagen, the absorption of iron, and the functioning of the immune system [11]. It is commonly added to food, beverages, medicines, and cosmetics [12, 13]. Both deficiency and overconsumption of AA are associated with health complications, thus necessitating the accurate detection of AA in biological and food samples [14]. The creation of sensors that are both sensitive and selective for the detection of AA is thus of great importance, particularly when combined with multifunctional nanomaterials capable of simultaneous antimicrobial action [15, 16].

Although nanostructures based on NCDs and Ag-NCDs have been previously studied, the synthesis and application of CuO-NCD nanocomposites for dual use in AA detection and antimicrobial activity remain underexplored [17]. From this study, we further evaluated their electrochemical response to AA and antimicrobial efficacy against bacteria species (*Staphylococcus aureus*,* Salmonella typhi*,* Escherichia coli*,* Bacillus cereus*) and fungi species (*Candida albicans*). The synthesized CuO-NCDs demonstrated high sensitivity, rapid response, and effective microbial inhibition, offering a promising platform for multifunctional biomedical and sensing applications [18, 19].

## Materials and methods

###  Materials

The following chemicals were employed without further purification: citric acid (C₆H₈O₇, ~ 99%), urea (CH₄N₂O ~ 99%), copper nitrate trihydrate (Cu(NO₃)₂·3 H₂O, ~ 99%, UNI-CHEM), sodium hydroxide (NaOH, 99%, Sigma-Aldrich, India), ammonia solution (NH₃, 28%), ascorbic acid (C₆H₈O₆, ~ 99), Potassium chloride (KCl, ≥ 99.5%), potassium ferricyanide (K₃[Fe(CN)₆], ≥ 99%), potassium ferrocyanide (K₄[Fe(CN)₆]·3 H₂O, ≥ 99%), monobasic potassium phosphate (KH₂PO₄ ~99%), dibasic potassium phosphate (K₂HPO₄ ~99%), uric acid (C₅H₄N₄O₃ ~99%), dopamine hydrochloride (≥ 98%), ethanol (CH₃CH₂OH, ~ 99%), dimethyl sulfoxide (DMSO, ≥ 99.9%, Sigma-Aldrich, USA), gentamicin (≥ 98%, Himedia, India), Mueller–Hinton agar (MHA), clotrimazole (≥ 99%, Sigma-Aldrich, USA), acetic acid (CH₃COOH), chitosan (C₅₆H₁₀₃N₉O₃₉), and hydrochloric acid (HCl, ~ 99%).

### Synthesis of nitrogen-doped carbon Dots (NCDs)

Nitrogen-doped carbon dots (NCDs) were prepared by a modified hydrothermal method [20]. A mixture of 1.0 g of citric acid and 0.5 g of urea was dissolved in 20 mL of distilled water. The solution was heated in a microwave oven at 200 °C at 700 W for 20 min. The solid product was dissolved in 20 mL of distilled water, then filtered and centrifuged at 4000 rpm for 30 min to eliminate larger particles. The microwave energy intensity was approximately 35 W/mL, ensuring reproducibility of the synthesis conditions.

### Synthesis of CuO nanoparticles

CuO NPs were synthesized by using a modified precipitation method [21, 22]. Briefly, 0.1 M of Cu(NO₃)₂·3 H₂O was dissolved in 100 mL of distilled water under stirring. Separately, 0.1 M NaOH solution was prepared and added dropwise to the copper solution to adjust the pH to 9, resulting in the formation of a blue precipitate. The resulting precipitate was filtered, washed four times with distilled water and ethanol, and then dried at 60 °C for 4 h to obtain CuO NPs.

###  Synthesis of CuO-NCD nanocomposites

CuO-NCD nanocomposites were synthesized based on previously reported methods with slight modifications [23–25]. Briefly, CuO NPs (400 mg) were dispersed in 50 mL of distilled water and sonicated for 30 min to obtain a uniform suspension. Then, 5 mL of NCD solution was added to the CuO NPs suspension, mixture was stirred continuously for 6 h to ensure proper interaction and composite formation. The resulting product was collected by centrifugation at 4000 rpm for 40 min, washed with ethanol to remove impurities, and dried at 80 °C for 2 h. The dried CuO-NCD NC powder was then used for further characterization and sensor fabrication.

###  Characterization techniques

Electrochemical assessments were performed utilizing a voltammetric analyzer (Epsilon EC-Ver 1.40.67, Bioanalytical System, USA) arranged in a standard three-electrode configuration: an unmodified or modified glassy carbon electrode (GCE) serving as the working electrode, an Ag/AgCl reference electrode, and a platinum wire functioning as the counter electrode. UV-Vis spectra were collected with a UV-Vis spectrophotometer (JENWAY 6705). X-ray diffraction (XRD) analysis was performed using a DR AWELL XRD-700 diffractometer. Fourier-transform infrared (FT-IR) spectroscopy measurements were carried out using a PerkinElmer spectrometer. The structure of the samples was examined using scanning electron microscopy (SEM, JCM-6000Plus).

###  Electrochemical sensor fabrication and testing

####  Preparation of supporting electrolyte

A phosphate buffer saline (PBS) with a concentration of 0.1 M and a pH range of 2 to 8 was created by mixing 6.8 g of KH₂PO₄ and 8.7 g of K₂HPO₄ in distilled water. To achieve the desired pH, adjustments were made using 1 M HCl and NaOH [26].

####  Preparation of standard solutions

A 1 mM stock solution of AA was prepared in 0.1 M PBS. Working solutions of varying concentrations were obtained by serial dilution [27].

####  Preparation of electrodes

The glassy carbon electrode (GCE) was sequentially polished using 0.3 μm alumina slurry, rinsed with distilled water, sonicated to eliminate residual alumina, and allowed to air-dry. Following a modified procedure based on literature, 0.027 g of each nanomaterial (NCD, CuO, and CuO-NCD) was prepared in a water–ethanol mixture (v/v = 1:1) containing 0.1% chitosan to enhance adhesion to the GCE surface. The solvent ratio was optimized to ensure uniform film formation and reproducibility of electrode preparation. Subsequently, 5 µL of each suspension was drop-casted onto the GCE and dried at room temperature for 24 h [28].

#### Optimization of experimental parameters

The pH effect on AA detection was studied in PBS (pH 2–8) employing cyclic voltammetry (CV) at a scanning rate of 100 mV·s⁻¹. The pH that produced the highest oxidation peak current was chosen for additional examination.

####  Selectivity study

Selectivity was evaluated by introducing common interfering metal ions and antioxidant species into a 1 mM AA solution. The corresponding changes in oxidation current were monitored and compared with the AA response.

####  Real sample analysis

Orange juice and vitamin C tablet samples were used to assess practical performance. Orange juice was obtained by pressing and centrifuging fresh fruits. Vitamin C tablets were powdered, and 0.02 g was dissolved in 100 mL PBS. A 5 µL aliquot was further diluted in 5 mL PBS for Cyclic voltametry (CV) analysis. Each sample was analyzed in triplicate [27]. The recovery percentage was determined using the subsequent Eq. (1):

1 $$ \begin{gathered} \% ~{\text{Recovery}}~ \hfill \\ = \frac{{{\text{Concentration}}{\mkern 1mu} ~{\text{of}}~{\mkern 1mu} {\text{spiked}} - {\text{Concentration}}~{\mkern 1mu} {\text{unspiked}}}}{{{\text{Concentration}}~{\mkern 1mu} {\text{of}}{\mkern 1mu} ~{\text{added}}{\mkern 1mu} ~{\text{amount}}}}~ \times ~100 \hfill \\ \end{gathered} $$

###  Antimicrobial activity assay

Antibacterial and antifungal tests were conducted at the Department of Biology, Jimma University. Mueller–Hinton agar was used to culture *S. typhi*, *B. cereus*, *S. aureus*, *E. coli* and *C. alibcans*. Each strain was subcultured on nutrient agar plates and incubated at 37 °C for 24 h to obtain fresh active cultures. The bacterial suspensions were then standardized to approximately 1.5 × 10⁸ CFU/mL (0.5 McFarland standard) using sterile saline solution. Discs (6 mm) soaked in nanomaterial solutions at concentrations ranging 12 to 200 mg/mL in dimethyl sulfoxide (DMSO) were placed onto the inoculated plates. Gentamicin and clotrimazole were used as positive controls for evaluating antibacterial and antifungal activities, respectively. Plates inoculated with bacteria were incubated at 37 °C for 24 h, while plates with fungi were incubated at 27 °C for 48 h [2]. After incubation, the zones of inhibition around the discs were measured in millimeters to assess antimicrobial efficacy. The inactivation of bacterial strains was determined by the clear zones indicating growth inhibition surrounding the nanomaterial-loaded discs [29].

##  Results and discussions

###  Structural and morphological characterization

NCDs were produced through a straightforward microwave-assisted technique utilizing urea and citric acid as the sources of carbon and nitrogen, respectively. A mixture of urea and citric acid was exposed to microwave irradiation at 700 W for 20 min. The solution turned dark brown upon heating, showing the creation of carbon dots, as documented in existing research [30].

CuO NPs were prepared by dissolving Cu(NO₃)₂·3 H₂O in distilled water, followed by the incorporation of NaOH, which changed the solution from blue to deep blue, suggesting the formation of a copper complex [31]. Upon heating, a black precipitate formed, indicating the formation of CuO NPs.

For CuO-NCD NCs, NCDs were added during the synthesis process. The functional groups in NCDs acted as agents for capping and reduction, preventing agglomeration and promoting the formation of stable CuO-NCD NCs. The effective synthesis of CuO nanoparticles (CuO NPs), nitrogen-doped carbon dots (NCDs), and the incorporation of NCDs into CuO nanoparticles was confirmed utilizing various characterization techniques including UV–Vis spectroscopy, FT-IR, XRD, and SEM [32]. Each provided complementary insights into the structural, optical, and electrochemical properties of the synthesized nanocomposites.

#### UV–Vis absorption and band gap determination

**Fig. 1:**
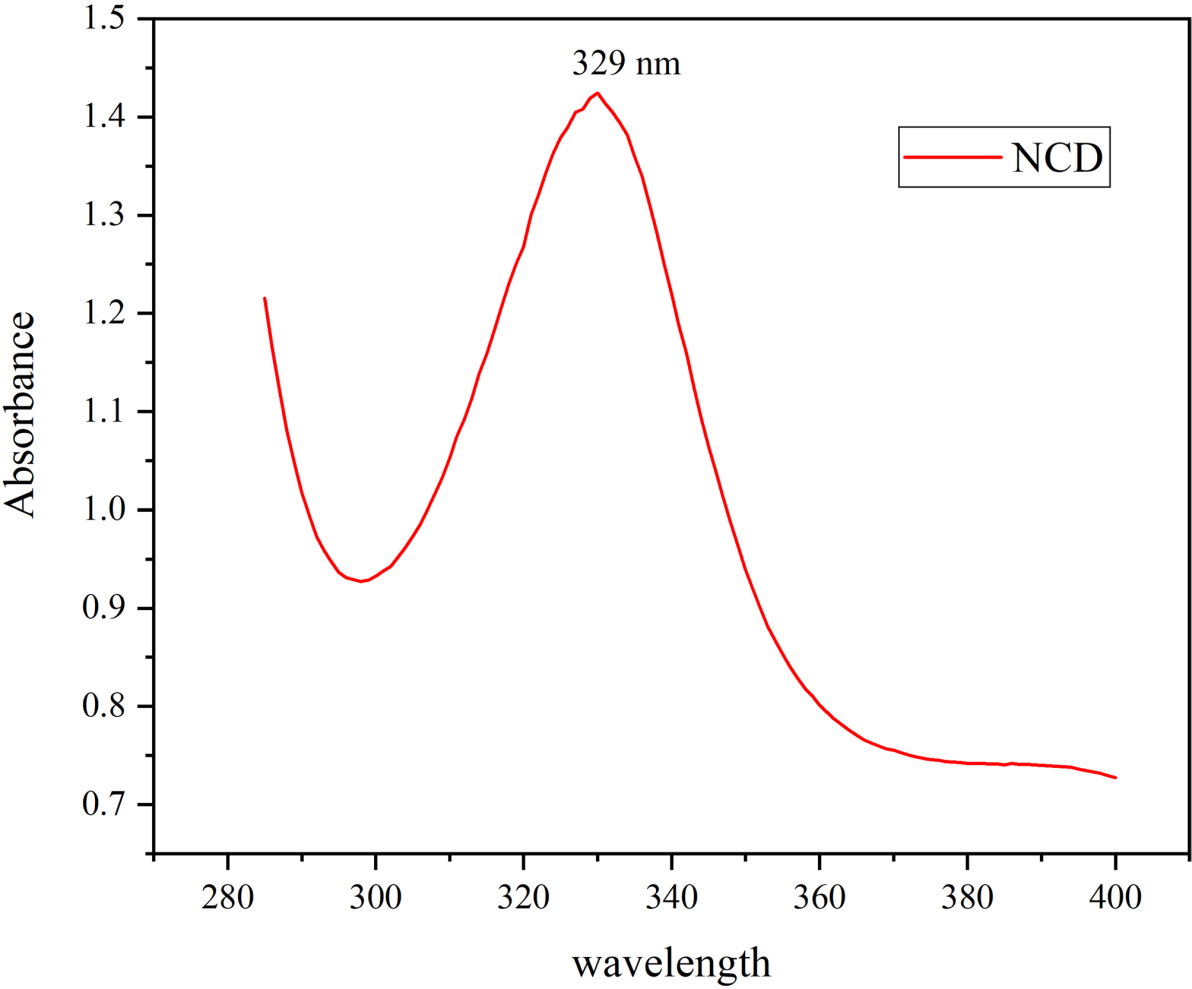
UV-Vis Absorption spectra of NCD recorded in aqueous solution

The optical properties of CuO NPs, NCDs, and CuO-NCD NCs were investigated using UV–Vis spectroscopy. NCDs show a strong absorption peak at 329 nm (Fig. 1) attributed to n–π* electronic transitions of surface carbonyl (C = O) groups.

**Fig. 2:**
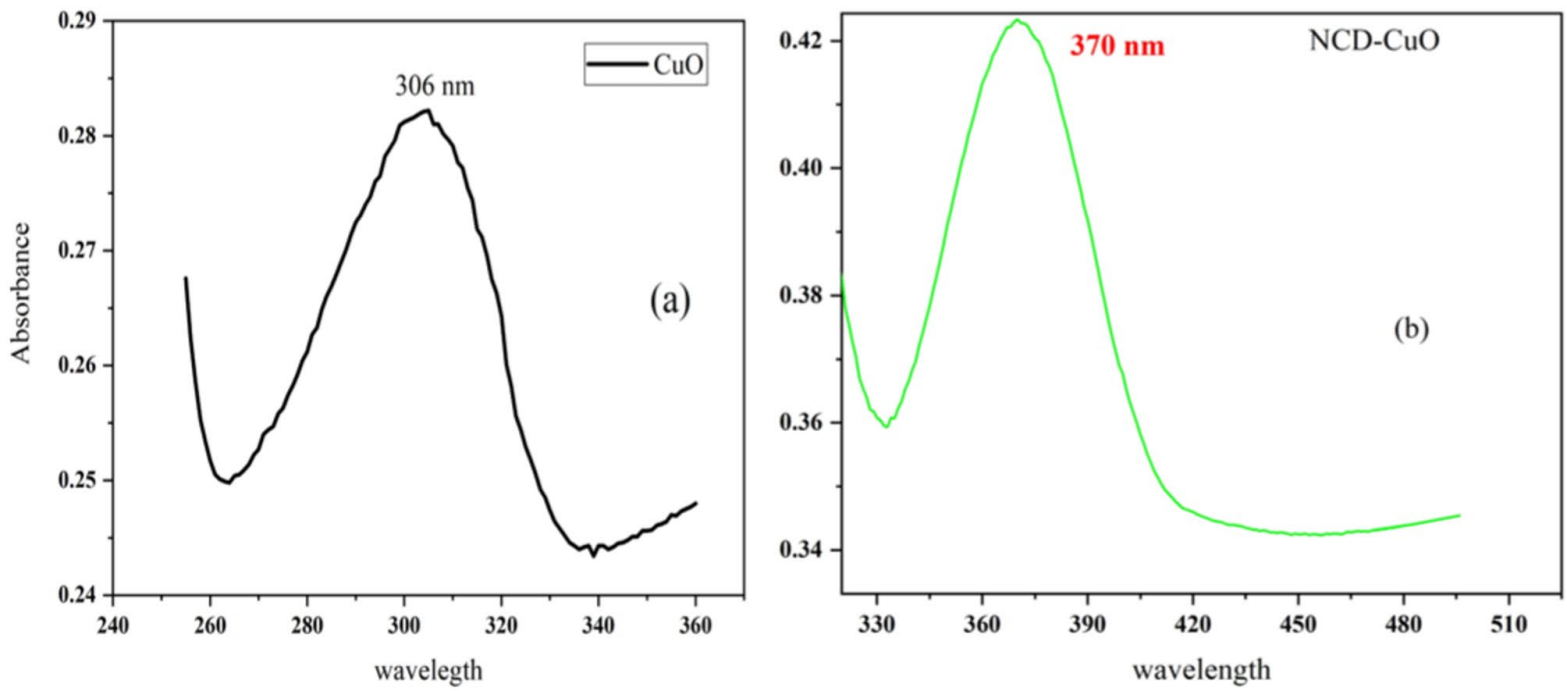
UV-Vis Absorption spectra of CuO NPs (**a**) and CuO-NCD NCs (**b**), showing characteristic absorption features and the effect of NCD incorporation

As shown in Fig. 2a, CuO nanoparticles display a characteristic absorption peak at 306 nm, in agreement with a literature report [33]. This band could be due to charge transfer transitions between O 2p and Cu 3d orbitals for CuO. The CuO-NCD NCs showed a redshifted absorption peak at 370 nm Fig. 2b), indicating successful composite formation [34]. This shift is likely due to interactions between CuO NPs and the functional groups that contain oxygen on the surface of the NCDs [35]. Moreover, the narrow and sharp nature of the peaks indicates the presence of smaller nanoparticles with good dispersion and minimal aggregation [36].

**Fig. 3:**
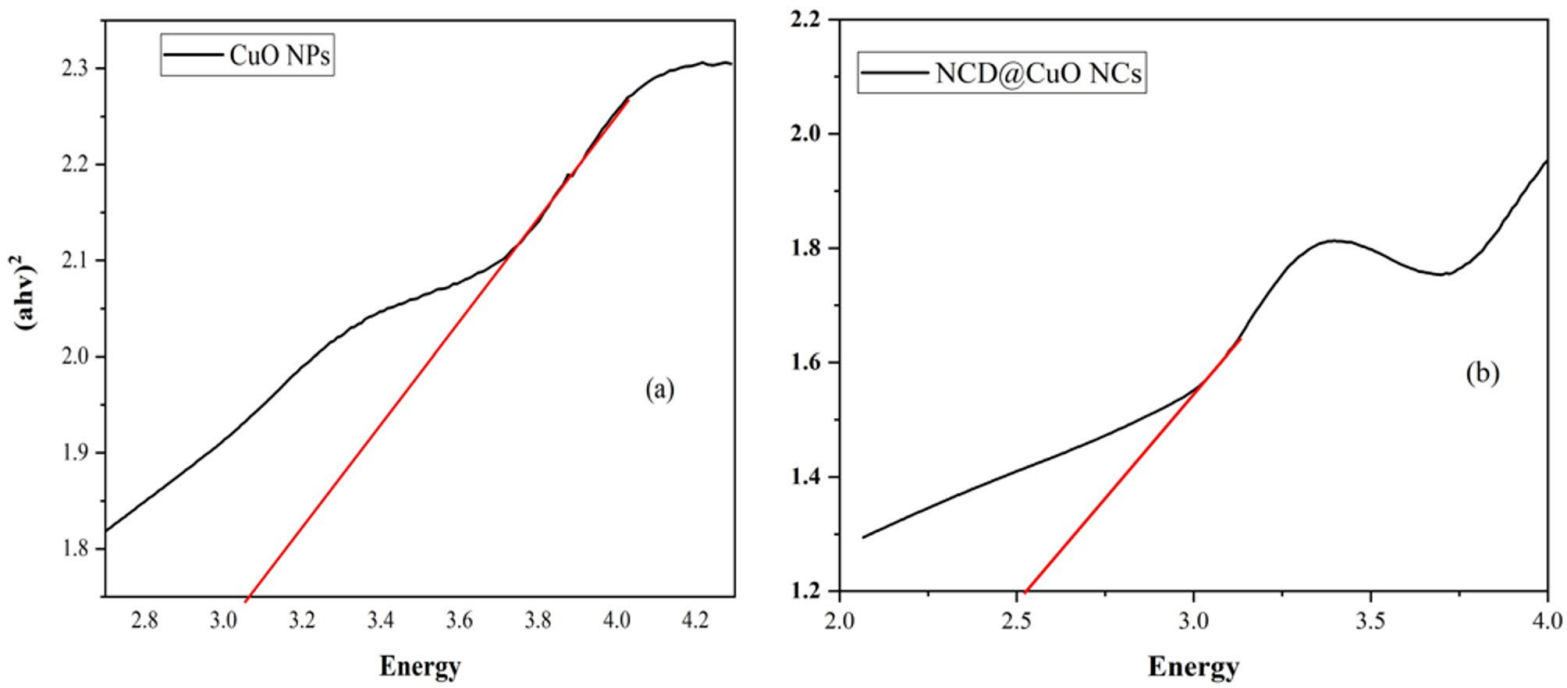
Tauc plots showing the calculated band gaps of CuO NPs (**a**) and CuO-NCD NCs (**b**)

The energies of the band gaps were determined through the use of Tauc plots (Fig. 3). The band gap analysis (Fig. 3) further supported the nanocomposite formation. CuO NPs had a calculated band gap of 3.05 eV (Fig. 3a), which exceeds that of bulk CuO (1.5 eV), indicating the quantum confinement effect in nanoscale structures [37]. In contrast, CuO-NCD NCs exhibited a lower band gap of 2.6 eV (Fig. 3b), attributed to an increased particle size and the formation of the nanocomposite structure [38]. The reduced band gap of CuO–NCD nanocomposites indicates strong electron transfer interactions between NCDs and CuO, facilitated by electrostatic attraction between opposite charges. This red shift in the absorption edge confirms effective adhesion of NCDs onto CuO nanoparticles, which may enhance antimicrobial and electrochemical performance [39].

#### FT-IR spectral analysis

**Fig. 4:**
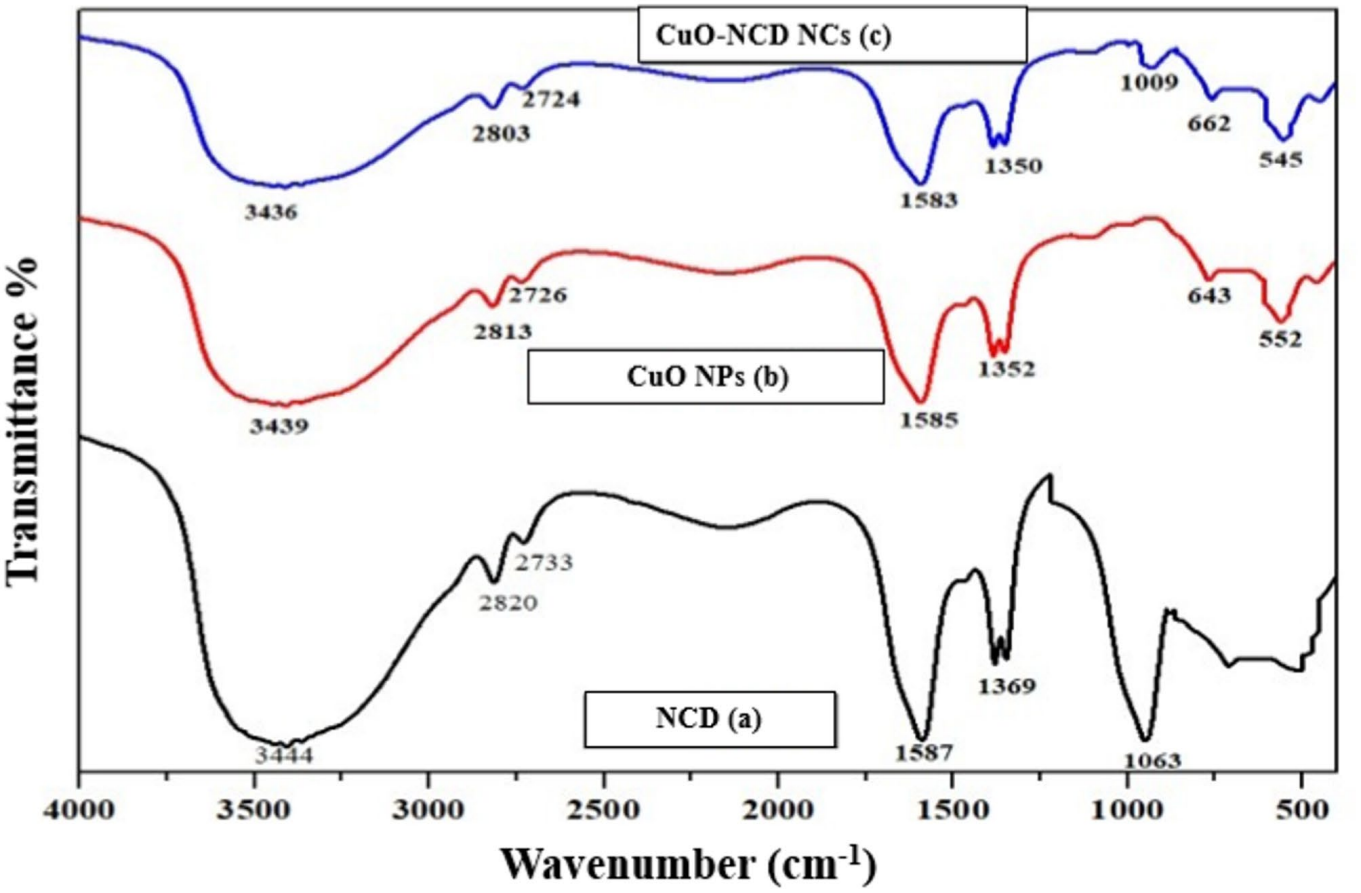
FT-IR spectra of NCD (**a**), CuO NPs (**b**), and CuO-NCD NCs (**c**), showing characteristic functional groups and interactions

 Figure 4 presents molecular-level evidence of nanocomposite formation, which was recorded between 4000 and 400 cm⁻¹. The broad absorption bands at 3200–3500 cm⁻¹ were assigned to the O–H and N–H stretching vibrations, indicative of surface hydroxyl groups arising from hydrogen bonding and amine groups present on the NPs and NCs [40]. In CuO-NCD NCs, the N–H/O–H peak shifted from 3444 to 3436 cm⁻¹ with decreased peak intensity in CuO-NCD NCs compared to NCDs, suggesting successful interaction through hydrogen bonding and coordination, reduced electron density, and formation of CuO-NCD NCs. Functional groups in NCDs originate mainly from urea and citric acid, with a sharp peak at 1063 cm⁻¹ indicating the existence of C–C structures [41]. The FT-IR spectrum of CuO NPs shows characteristic absorption bands at 552 and 643 cm⁻¹, which are assigned to Cu–O bending and stretching vibrations within the monoclinic CuO lattice, confirming the formation of CuO. The low-wavenumber peaks (500–700 cm⁻¹) region confirms Cu–O bonding and the presence of CuO, consistent with previous reports [42].

#### XRD analysis

**Fig. 5:**
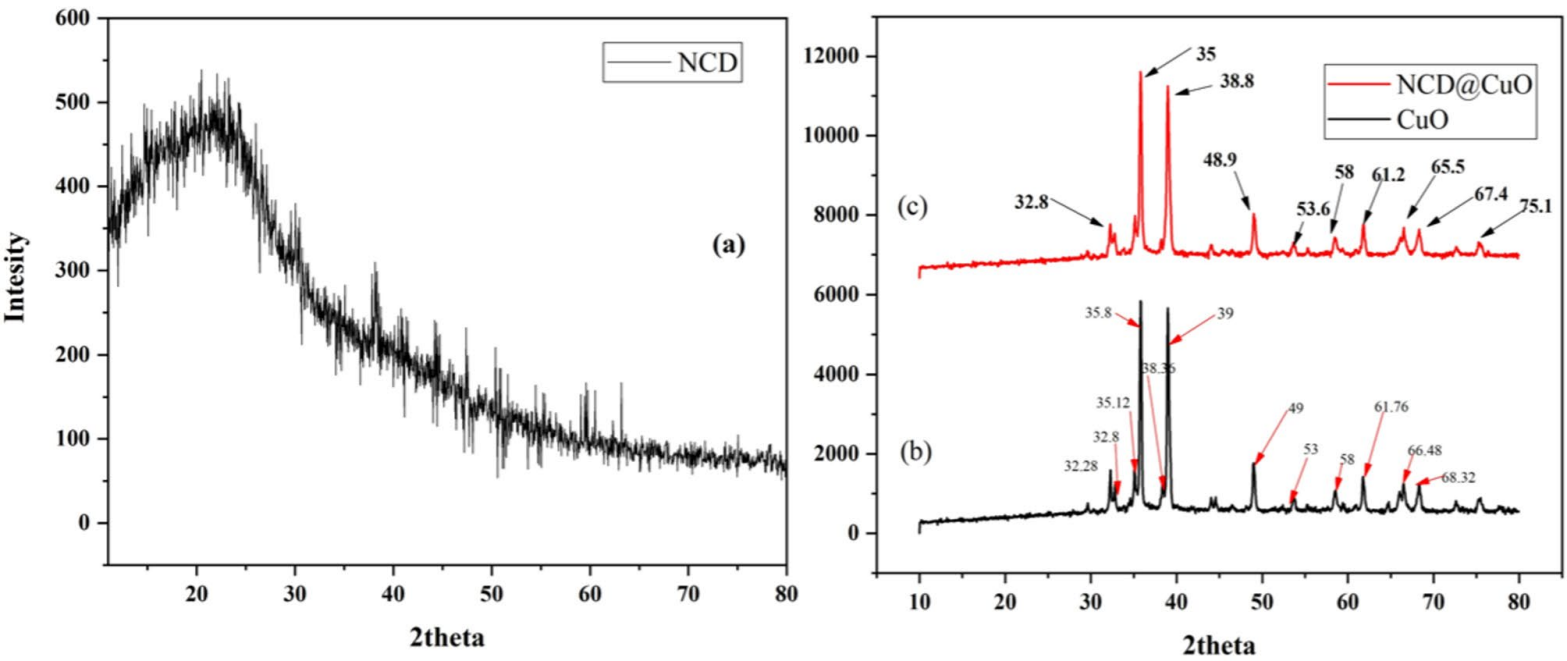
The XRD diffraction patterns of the synthesized NCDs (**a**), CuO NPs (**b**), and CuO-NCD NCs (**c**)

Figure 5 shows the XRD patterns of nitrogen-doped carbon dots (NCDs) (a), CuO NPs (b) and the CuO-NCDs NCs (c). XRD analysis confirmed the amorphous nature of NCDs, which display a broad peak around 2θ ≈ 23°, characteristic of amorphous carbon [43] (Fig. 5a). The CuO NPs show distinct diffraction peaks at 2θ = 32.28, 32.80, 35.12, 35.80, 39.00, 49.04, 53.68, 58.55, 61.76, 66.48, and 68.32°, corresponding to the (110), (111), (200), (202), (020), (202), (113), (310), (220), (311), and (222) planes of monoclinic CuO (JCPDS PDF No. 89-5895) [44] (Fig. 5b).

In the CuO–NCD NCs, the CuO diffraction peaks are slightly broadened and shifted, indicating strong interactions between CuO and NCDs and possible changes in crystallite size or lattice strain (Fig. 5c) [45]. The average crystallite size of CuO nanoparticles was calculated using the Debye–Scherrer Eq. (2) from the most intense peaks at 2θ = 32.8, 35, 38.8, 48.9, 53.6, 58.0, 61.2, 65.5, 67.4, and 75.1° in the XRD pattern [46]. The calculation yielded ~ 20 nm for CuO NPs and ~ 23 nm for CuO–NCD NCs, indicating a slight increase in size due to NCD embedding.

2 $$\:\text{D}=\frac{\text{k}{\uplambda\:}\:}{{\upbeta\:}\text{h}\text{k}\text{l}\:\text{C}\text{o}\text{s}\:{\uptheta\:}}$$

#### SEM analysis

**Fig. 6:**
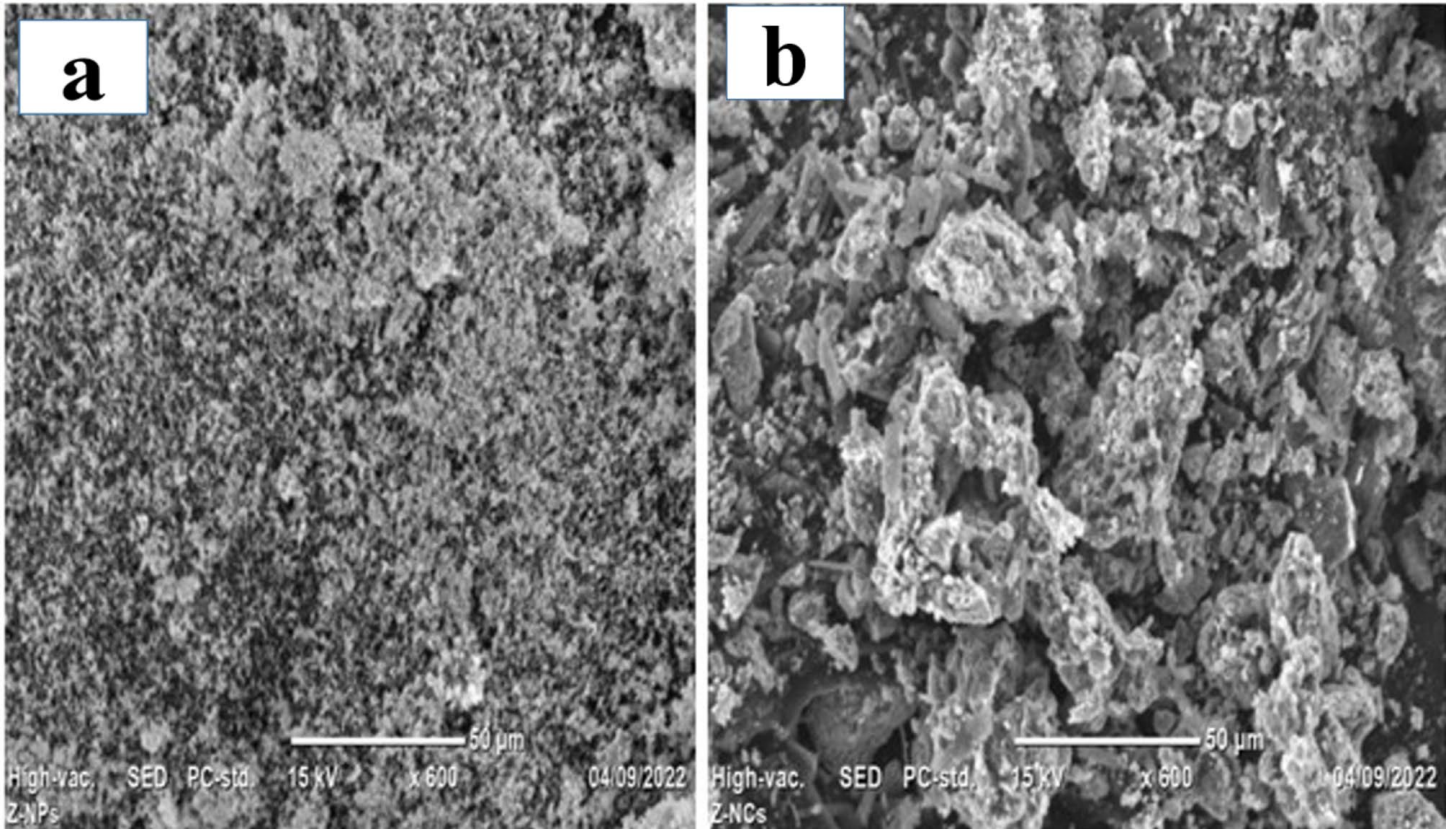
SEM data of CuO NPs (**a**) and CuO-NCD NCs (**b**), showing morphology and particle size distribution

**Fig. 7:**
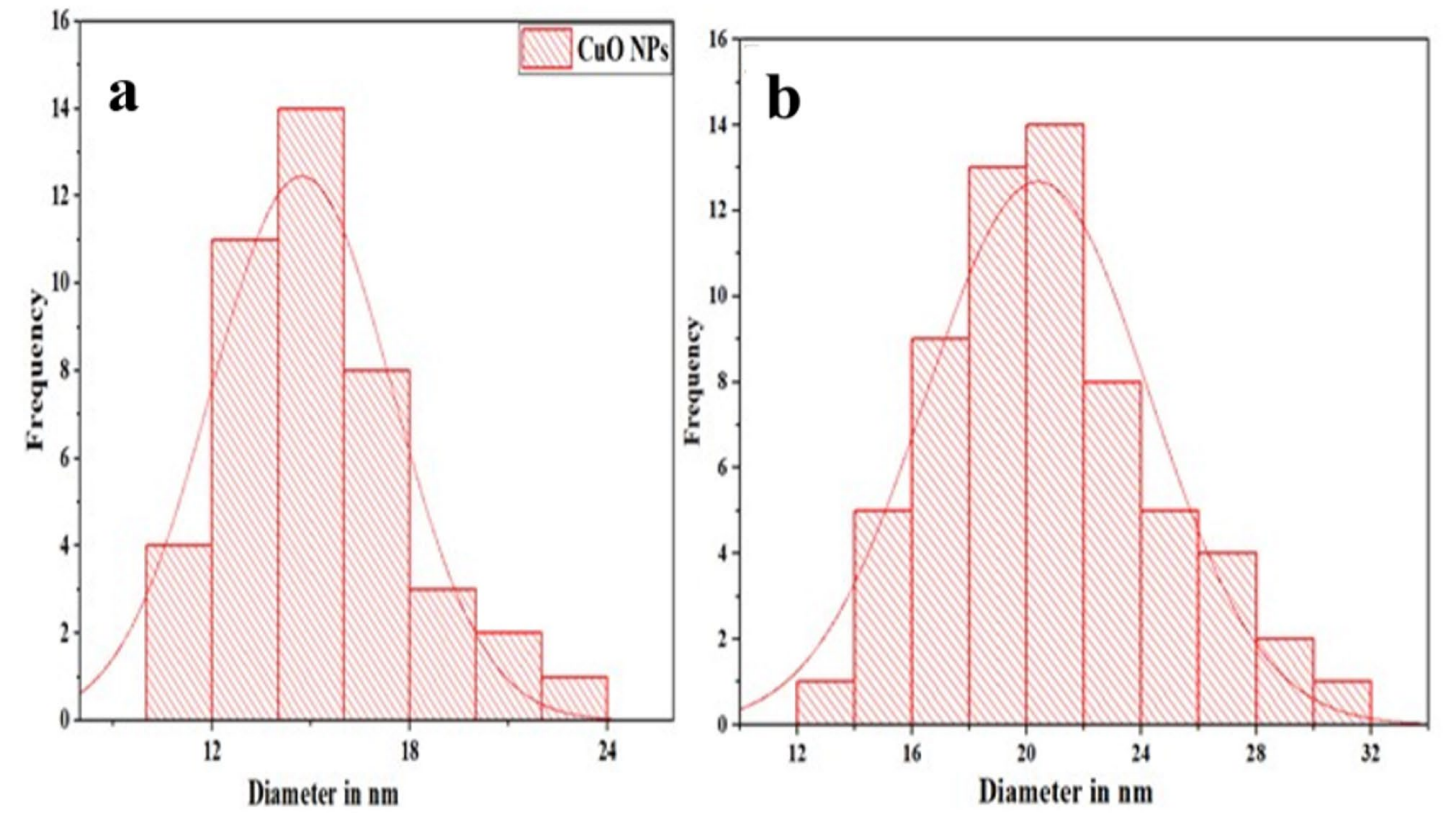
Histogram showing the particle size distribution pattern for the SEM images of CuO NPs (**a**) and CuO-NCD NCs (**b**)

SEM images (Fig. 6) revealed spherical morphologies for CuO and CuO-NCDs. CuO NPs displayed uniformly distributed particles and spherical NPs (Fig. 6a). CuO-NCD NCs appeared agglomerated and exhibited a heterogeneous mixture of rod-like and spherical particles (Fig. 6b), indicating morphological alteration resulting from the interaction with NCD [47]. This structural change enhances electrochemical surface area and conductivity. The increase in average particle size in CuO-NCD NCs was consistent with the band gap reduction and indicates successful integration of NCDs into the CuO matrix. The average particle size distributions were determined by measuring from SEM images using ImageJ software. The particle sizes were plotted as a histogram (Fig. 7), illustrating that the average particle size distribution pattern of CuO NPs (Fig. 7 (a)) was smaller than that of CuO-NCD NCs (Fig. 7b).

#### Zeta potential measurements

The NCD–CuO nanocomposite demonstrated a zeta potential of + 26 mV, surpassing that of pristine NCD (+ 22.46 mV) and CuO nanoparticles (+ 17.57 mV). This rise in surface charge indicates enhanced electrostatic repulsion among particles, which can improve colloidal stability and facilitate better dispersion in aqueous environments for electrochemical and antimicrobial purposes.

### Electrochemical performance

**Fig. 8:**
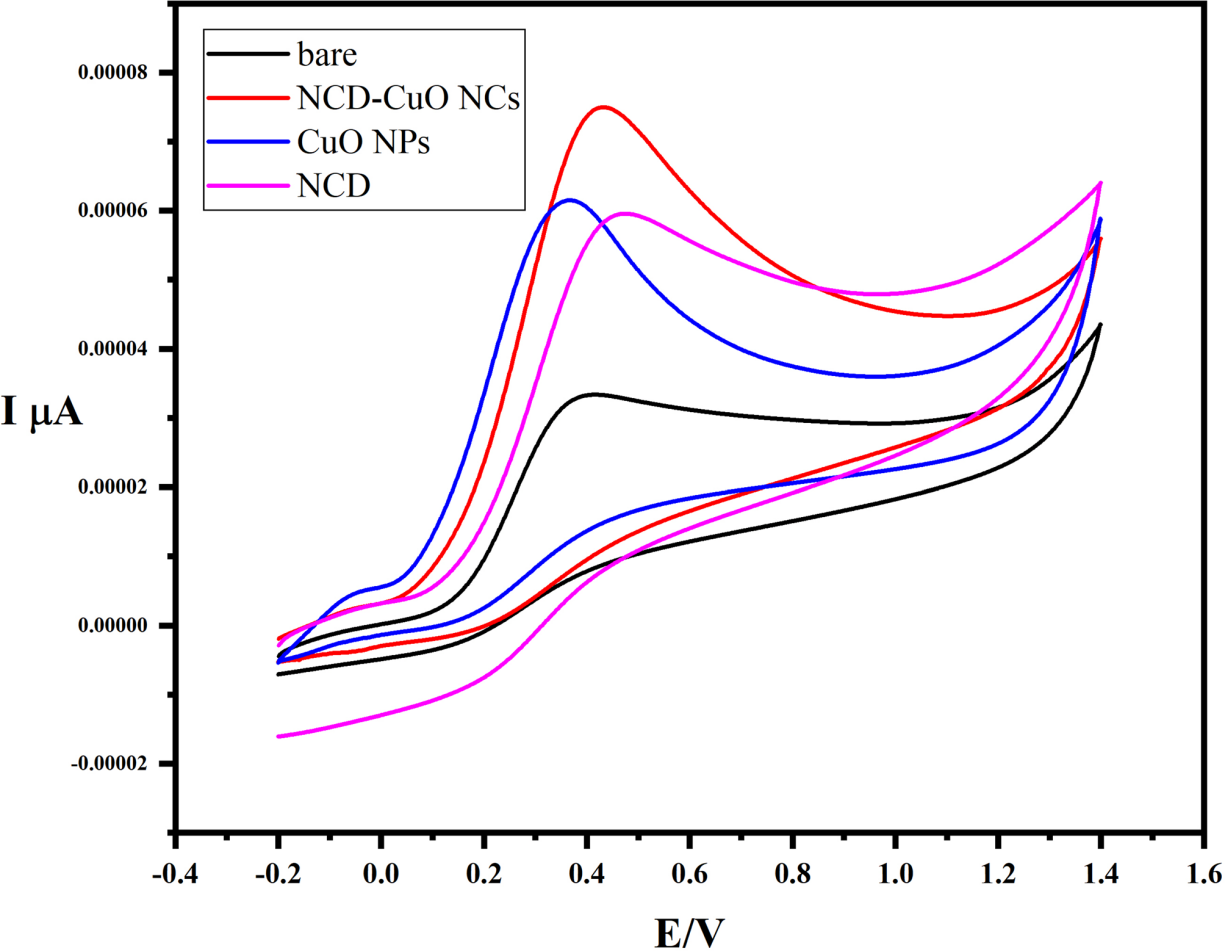
Comparison of CuO NPs and NCD-CuO NCs in PBS buffer and ascorbic acid at pH-3

The cyclic voltammetric responses of 1 mM ascorbic acid (AA) at the bare electrode, NCD, CuO, and CuO–NCD modified electrodes are shown in Fig. 8. The CuO–NCD nanocomposite exhibited the highest anodic peak current among all tested electrodes, indicating significantly enhanced electrochemical activity. This enhancement is attributed to the synergistic interaction between CuO and NCDs, which increases the effective surface area and improves electron transfer kinetics [48].

The oxidation peak potential of AA shifted toward negative values with increasing pH, confirming the involvement of protons in the electro-oxidation process at the CuO–NCD surface [49]. At higher pH values, the peak current decreased, likely due to reduced catalytic activity from fewer available protons and active sites [50]. The linear relationship between the oxidation peak current and the square root of the scan rate indicates a diffusion-controlled and irreversible process [51].

This behavior aligns with electrochemical theory and supports the efficient electrocatalytic capability of the CuO-NCD nanocomposite. The strong linearity and low detection limit confirm the high sensitivity of the CuO-NCD/GCE sensor toward AA detection. The sensitivity of the CuO–NCD/GCE electrode was calculated from the slope of the calibration curve (µA·µM⁻¹) normalized by the effective surface area (cm²), giving a value of 0.385 µA·µM⁻¹·cm⁻². The reproducible response across concentrations demonstrates good sensor stability and potential for quantitative applications [52].

The minimal change in the oxidation current in the presence of interferents indicates that the sensor maintains strong selectivity for AA. This is essential for real-sample analysis where multiple electroactive species may coexist [53]. The sensor exhibits moderate stability over time. The gradual decline in current response may be due to surface fouling or partial degradation of the active material, but performance remains acceptable for short- to medium-term applications. The high recovery rates confirm the practical applicability of the CuO-NCD sensor in real sample matrices. The sensor effectively detects AA in complex samples without significant interference from other substances.

The sensitivity of the CuO–NCD/GCE electrode was calculated from the slope of the calibration curve normalized by the effective surface area, resulting in 0.385 µA·µM⁻¹·cm⁻². The limit of detection (LOD) was determined using the formula LOD = 3σ/S, where σ is the standard deviation of the blank signal and S is the slope of the calibration curve. This yielded an LOD of 2.56 µM, demonstrating the sensor’s ability to detect low concentrations of AA. The sensor displayed a linear detection range of 5–120 µM, showing superior sensitivity compared to CuO nanoparticle-modified carbon paste electrodes (LOD = 5 µM, linear range = 10–800 µM) [23] and N-doped carbon dot-modified GCEs (LOD = 4.5 µM, linear range = 10–150 µM) [54]. The enhanced performance is attributed to the combination of high conductivity from NCDs and electrocatalytic activity of CuO, which provides more active sites and facilitates faster electron transfer [55]. The reproducibility of the response across concentrations demonstrates good sensor stability, while minimal changes in current in the presence of common interferents confirm excellent selectivity for AA. The sensor also showed satisfactory performance in real sample matrices, indicating practical applicability.

#### Surface area of the electrodes

The effective surface area of the glassy carbon electrode (GCE) was evaluated before and after it was modified with the CuO-NCD nanocomposite using cyclic voltammetry with a 5 mM [Fe(CN)₆]³⁻/⁴⁻ redox probe. The surface area was determined using the Randles–Sevcik equation. The modified electrode exhibited an increased surface area of 0.12 cm², as opposed to 0.07 cm² for the unmodified GCE, suggesting that the nanocomposite coating provided more electroactive sites [56].

####  Effect of pH

**Fig. 9:**
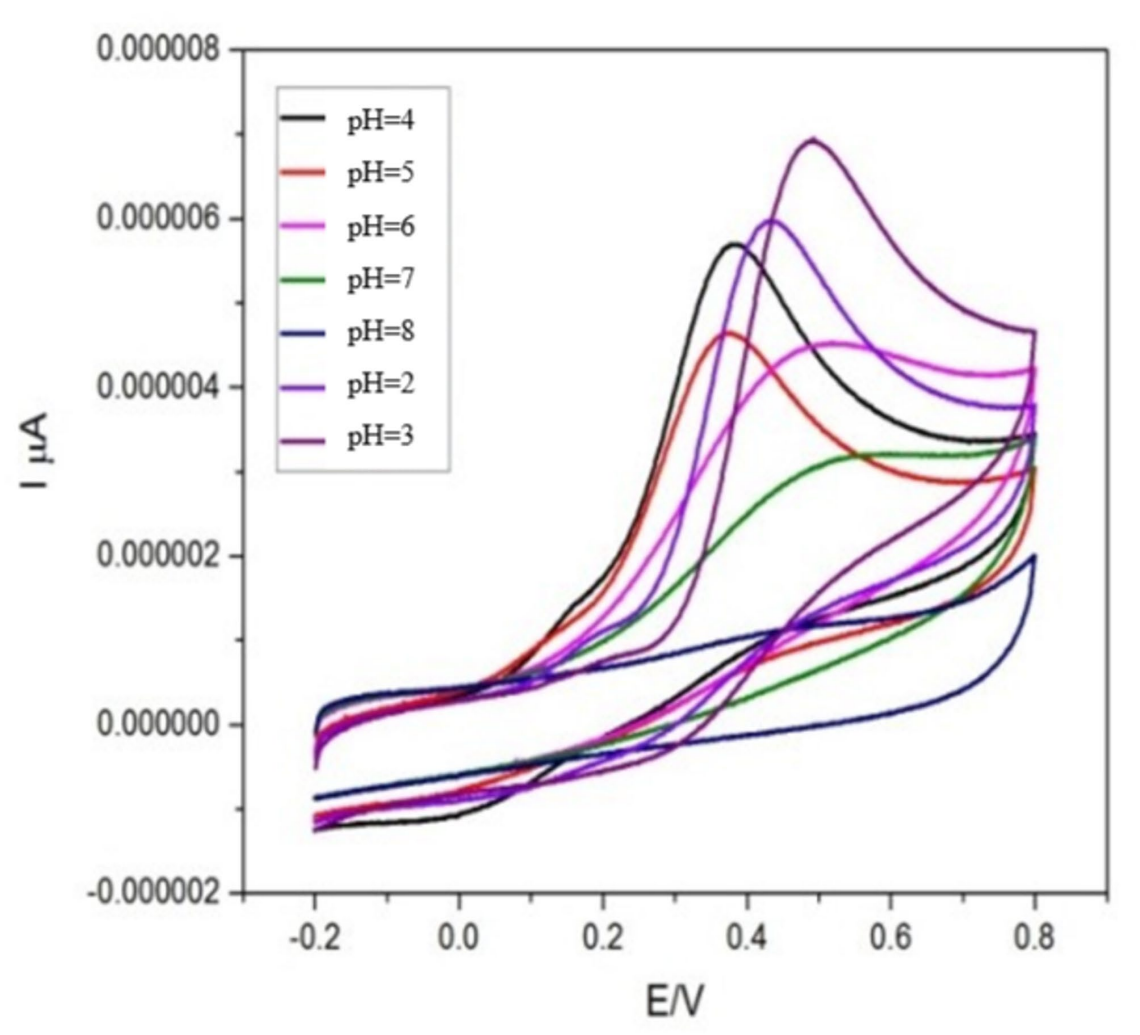
Effect of pH on the electrochemical response of CuO–NCD nanocomposites, showing current variations at different pH values

The electrochemical oxidation of ascorbic acid was investigated over a pH range of 3.0 to 8.0 (Fig. 9), and the maximum anodic peak current with the most defined voltammetry response was observed at pH 3.0. This acidic condition was therefore chosen as the optimum working environment. The enhanced performance at pH 3.0 can be attributed to more efficient proton-coupled electron transfer, which facilitates faster electron kinetics of AA oxidation.

Beyond improving detection sensitivity, operation at pH 3.0 also offers important advantages: it maintains the structural stability of the CuO–NCD nanocomposite, prevents degradation that may occur in alkaline conditions, and reduces interference from competing electroactive species that are more active at higher pH values. Hence, pH 3.0 was selected as the most suitable medium for achieving both analytical reliability and sensor stability [57].

#### Effect of scan rate

**Fig. 10:**
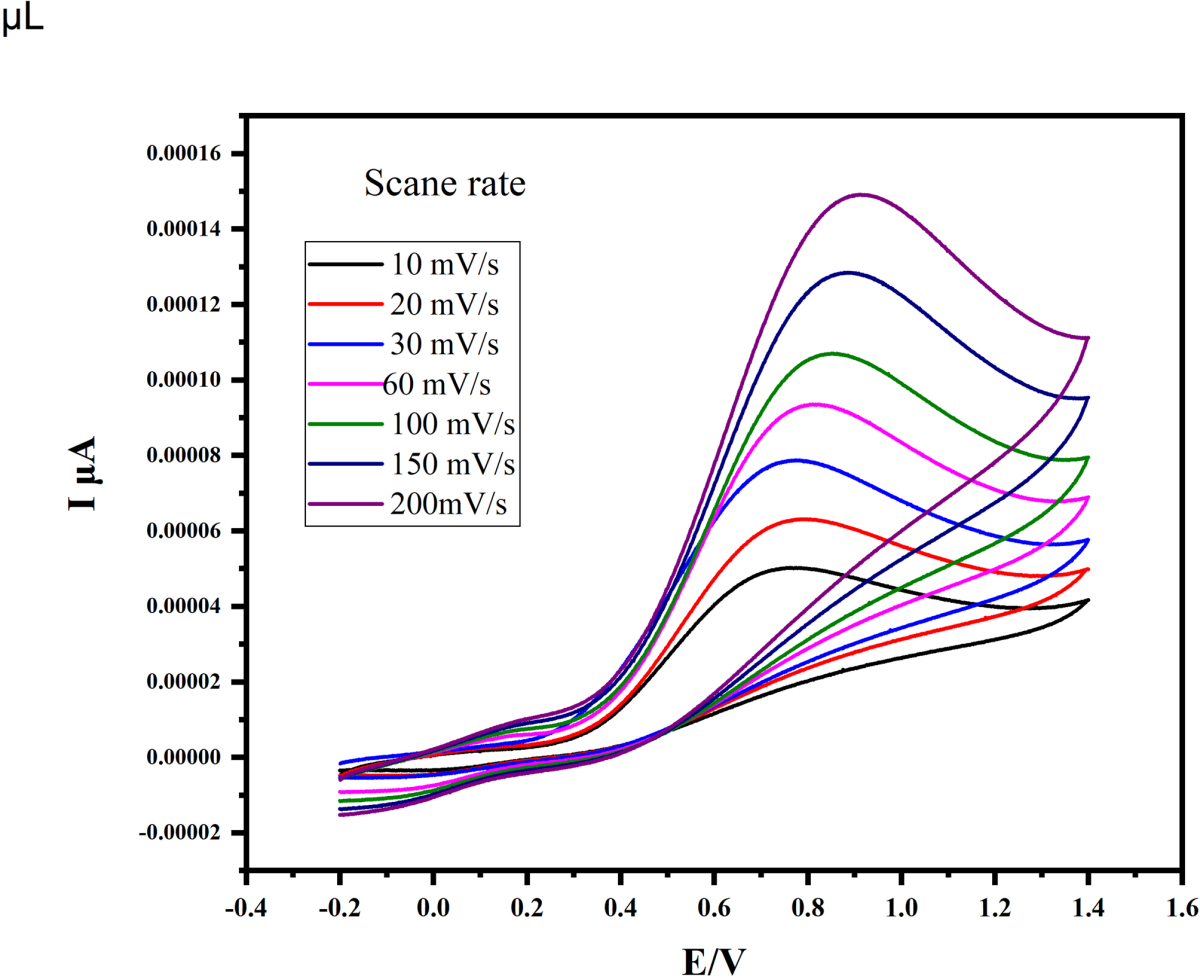
Effect of scan rate (10–200 mVs^− 1^) on the cyclic voltammetry response of CuO–NCD nanocomposites

Figure 10 displays the cyclic voltammetry of AA at scan rates ranging from 10 to 200 mV·s⁻¹ (Fig. 10). The oxidation peak current increased with scan rate, and a shift toward more positive potential was observed. A linear correlation was observed between the square root of the scan rate and the oxidation peak current, with a correlation coefficient of R² = 0.9906 (Fig. 10), indicating diffusion-controlled electrochemical behavior [58].

####  Effect of concentration and sensitivity

**Fig. 11:**
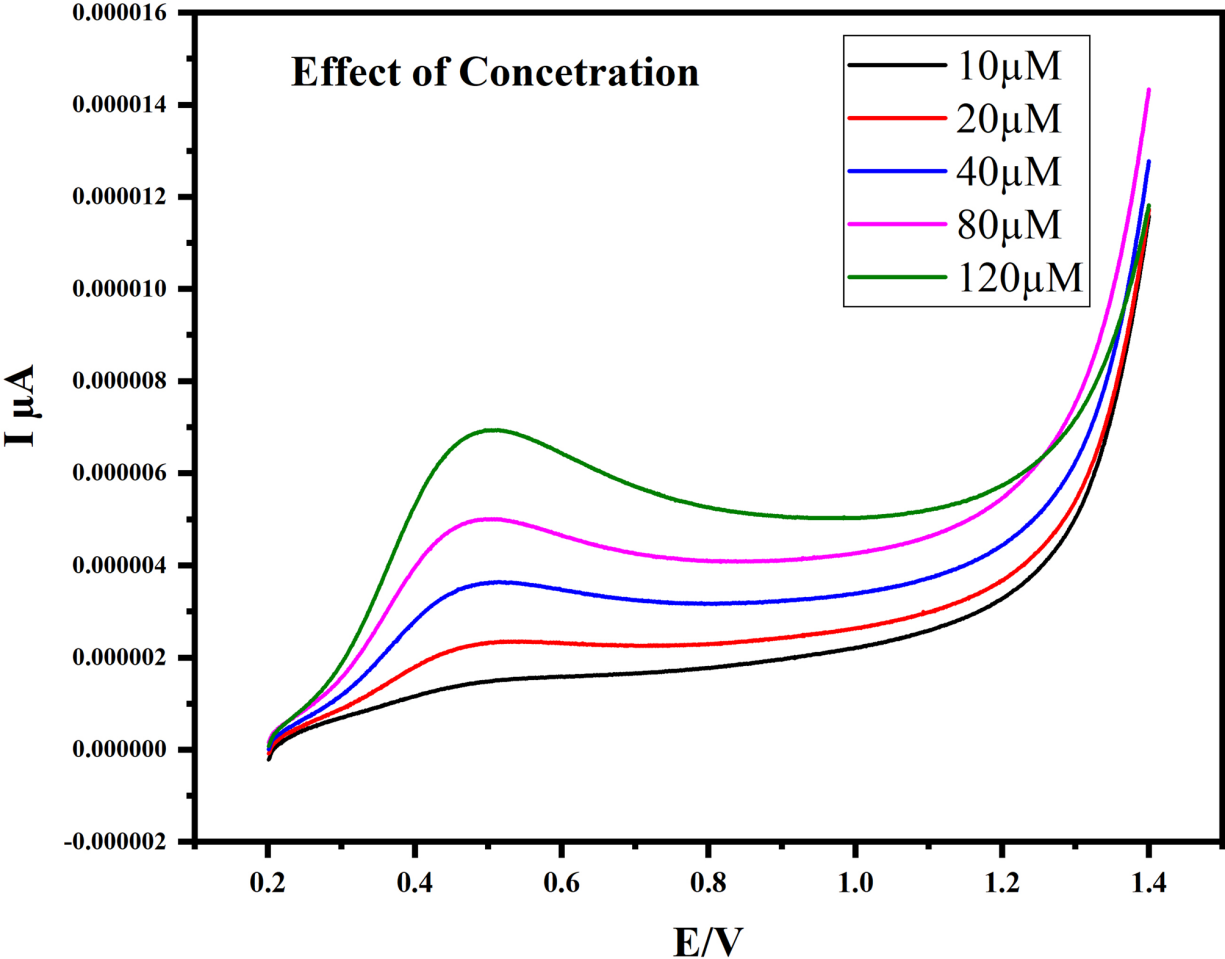
Calibration graph of different concentrations of ascorbic acid from (10–120 µM) in 0.1 M PBS using LSV

As illustrated in Fig. 11, increasing the AA concentration from 5 to 120 µM resulted in a proportional rise in peak current. The calibration plot followed the linear regression equation y = 0.0462x + 1.3844 with an R² value of 0.9914 (Fig. 11), confirming excellent linearity. The limit of detection (LOD) was calculated as 2.56 µM using the standard 3σ/m method, where σ is the standard deviation of the blank signal and *m* is the slope of the calibration curve.

From Table 1, the CuO–NCD nanocomposite sensor displayed a linear range of 5–120 µM with an LOD of 2.56 µM, which is superior to N-doped carbon dots/GCE (LOD = 4.5 µM) and CuO NPs/CPE (LOD = 5.0 µM). Its detection limit was also markedly better than microporous carbon (LOD = 23.1 µM) and slightly superior to the Bi₂S₃/rGO composite (LOD = 2.9 µM). These results indicate that the CuO–NCD sensor is highly suitable for trace-level detection applications, particularly in the low-µM range, owing to its excellent sensitivity and reliable performance.

**Table 1:** Performance comparison of the CuO–NCD sensor, including limit of detection (LOD, µM) and stability, with values reported in the literature

Sensor/material	Linear range (µM)	LOD (µM)	Ref.
CuO–NCD NCs	5–120	2.56	Present study
CuO NPs / CPE	10–800	5.0	[23]
N-doped carbon dots / GCE	10–150	4.5	[54]
Bi₂S₃/rGO composite	5-1200	2.9	[59]
Microporous carbon (MC/GCE)	100–2000	23.1	[60]

####  Stability of CuO-NCD NC sensor

Stability testing showed that the sensor retained approximately 71.4% of its initial current response after 9 days of storage at room temperature, indicating moderate stability under optimized conditions.

###  Selectivity

**Fig. 12:**
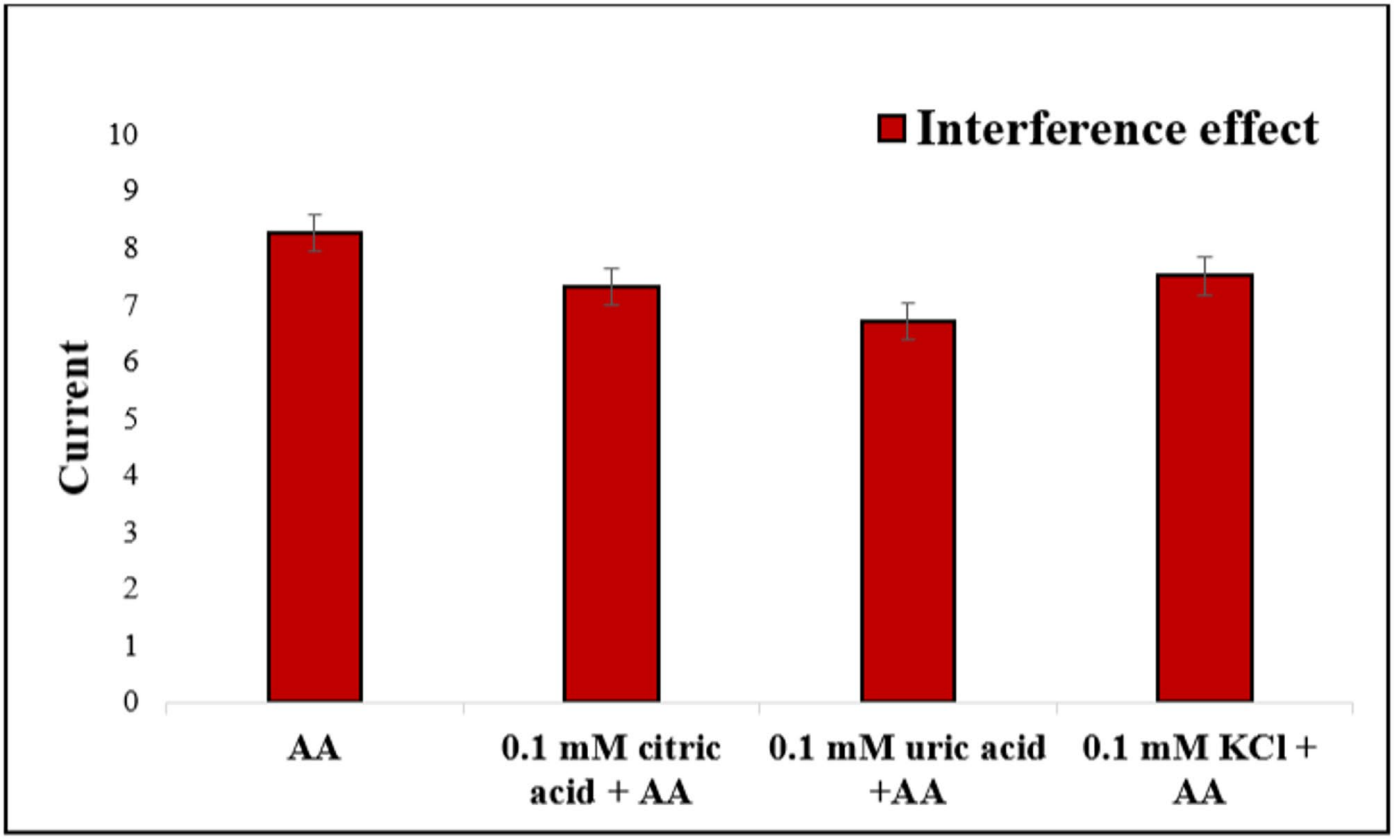
Current response of the CuO–NCD electrode to 1.0 mM AA alone and with 0.1 mM citric acid, uric acid, and KCl in orange juice (*n* = 3, mean ± SD): AA 8.27 ± 0.06; citric acid + AA 7.30 ± 0.10; uric acid AA 6.70 ± 0.10; KCl + AA 7.50 ± 0.10

The selectivity of the CuO-NCD modified electrode was examined in the presence of common coexisting species found in orange juice, including antioxidants (ascorbic acid, hesperidin), organic acids (citric acid), and common metal ions (Fe³⁺, Cu²⁺, Zn²⁺, Ca²⁺, Mg²⁺). As shown in Fig. 12, the addition of 1.0 mM AA (target analyte) produced a highest current response (8.27 ± 0.06 µA, *n* = 3). In contrast, the presence of 0.1 mM citric acid, uric acid, and KCl with AA resulted in slightly lower responses of 7.30 ± 0.10 µA, 6.70 ± 0.10 µA, and 7.50 ± 0.10 µA, respectively. These results, supported by error bars (mean ± SD), confirm that the CuO–NCD sensor retains excellent selectivity for AA detection even in the presence of potential interferents.

###  Real sample analysis

The CuO-NCD sensor was applied to determine AA in vitamin C tablets and orange juice using the standard addition method. Baseline measurements (0 µM added AA) were included to account for the naturally present AA in the samples.

As shown in Table 2, the sensor exhibited good accuracy, with recovery rates ranging from 92.99% to 109.45% and acceptable precision as indicated by the standard deviations and RSD values. These results demonstrate that the sensor is reliable for AA determination in complex matrices.

**Table 2:** Determination of AA in vitamin C tablets and orange juice by standard addition, showing mean ± SD, RSD (%), and recovery (%), with 0 µM representing native AA

Samples	Added AA (µM)	Founded AA (µM, mean ± SD)	RSD (*n* = 3%)	Recovery (%)
Vitamin C tablet	0	4.50 ± 0.14		
	5	4.59 ± 0.37	8.03	109.45
	10	9.64 ± 0.59	6.08	92.99
	20	19.61 ± 0.73	3.72	101.23
Orange juice	0	4.95 ± 0.17		
	5	5.43 ± 0.55	10.13	108
	10	9.39 ± 0.88	9.38	93.92
	20	20.19 ± 0.61	7.95	100.95

### Antimicrobial activity

**Fig. 13:**
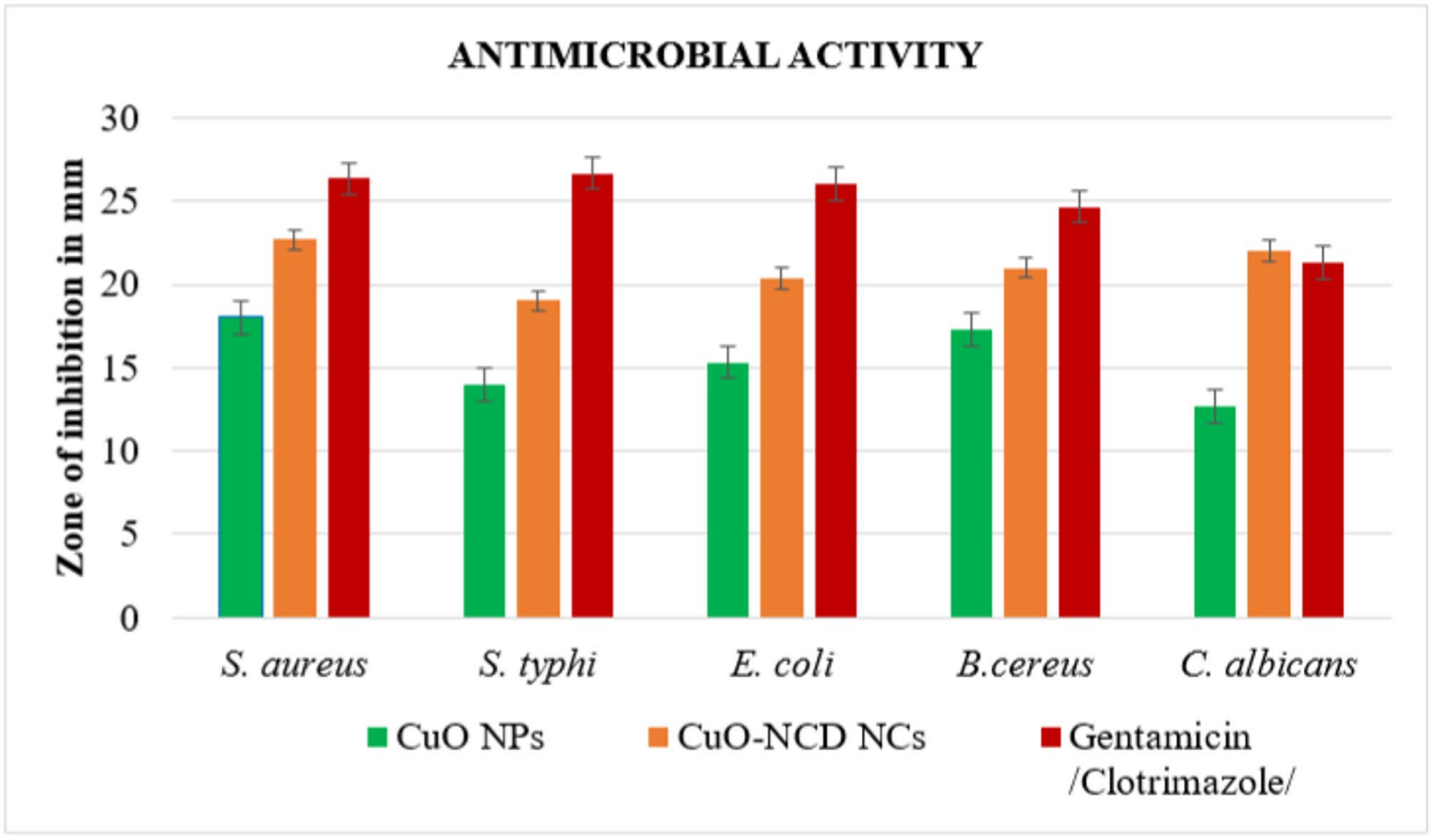
Histogram distribution pattern for antibacterial and antifungal species of CuO NPs and CuO-NCD NCs

The antibacterial properties of NCD, CuO NPs, and CuO-NCD NCs were evaluated against gram-negative and gram-positive bacteria using the disk diffusion method. CuO and CuO-NCD exhibited increasing inhibition zones with increasing concentration (12–200 mg/mL), while NCD showed minimal activity. CuO NPs at 200 mg/mL exhibited measurable zones of inhibition (Table 3), while CuO–NCD NCs showed enhanced antimicrobial performance, as illustrated by the histogram in Fig. 13. Overall, the results indicate that the incorporation of NCDs into CuO significantly improves the nanocomposite’s inhibitory effect against both bacterial and fungal species. The antimicrobial activity of the synthesized CuO–NCD NCs in this study is consistent with previous reports on biosynthesized CuO NPs. For example, CuO NPs synthesized using *Salacia reticulata* extract demonstrated significant inhibition against *S. aureus* and *E. coli*, with zones of inhibition up to 17 mm [61]. In comparison, our CuO–NCD NCs exhibited slightly higher inhibition zones (19–23 mm), suggesting that the incorporation of NCDs enhances the antimicrobial efficacy of CuO NPs, likely due to increased surface area and improved interaction with microbial cell walls.

**Table 3:** Zone of Inhibition of CuO NPs and CuO-NCDs NCs against different bacterial and fungal strains at 200 mg/mL

Species/sample	Zone of inhibition of bacterial and fungal species in mm				
	*S. aureus*	*S. typhi*	*E. coli*	*B. cereus*	*C. albicans*
CuO-NCD NCs	22.67 ± 0.58	19.00 ± 1.00	20.33 ± 0.58	21.00 ± 1.00	22.00 ± 1.00
CuO NPs	18.00 ± 1.00	14.00 ± 1.00	15.33 ± 0.58	17.33 ± 0.58	12.67 ± 0.58
Gentamicin /clotrimazole	26.33 ± 0.58	26.67 ± 0.58	26.00 ± 1.00	24.67 ± 0.58	21.33 ± 0.58

##  Conclusions

In this study, nitrogen-doped carbon dots and copper oxide nanoparticles were successfully synthesized from citric acid, urea, and copper nitrate using hydrothermal and precipitation methods, respectively. The synthesized nanomaterials were characterized for their structural and morphological properties using ultraviolet–visible spectroscopy, Fourier-transform infrared spectroscopy, X-ray diffraction, and scanning electron microscopy. Ultraviolet–visible spectroscopy showed a red shift in the absorption peak for the nanocomposites compared to the individual components, with calculated band gap energies of 3.05 eV for copper oxide nanoparticles and 2.6 eV for the nanocomposites. Fourier-transform infrared analysis confirmed the presence of functional groups responsible for surface capping and stabilization, while X-ray diffraction patterns indicated the crystalline nature and nanoscale dimensions of both nanoparticles and nanocomposites. Scanning electron microscopy images showed spherical and rod-like morphologies, with an increase in particle size for the nanocomposites.

Electrochemical studies demonstrated enhanced performance of the nanocomposite-modified glassy carbon electrode toward the detection of ascorbic acid. Cyclic voltammetry and linear sweep voltammetry results indicated that the nanocomposite-modified electrode exhibited a higher effective surface area and rate constant compared to other modified electrodes. Under optimized conditions (pH 3, applied potential + 0.4 V), the sensor showcased a linear detection range of 5–120 µM for AA with a detection limit of 2.56 µM. The sensor also demonstrated selectivity toward ascorbic acid when common interfering substances were present and retained 71% of its initial response after 9 days, indicating moderate stability. Furthermore, the nanocomposites exhibited strong antimicrobial effectiveness against various bacterial and fungal species, with particularly significant inhibition observed at higher concentrations. Overall, the results indicate that the synthesized nanocomposites possess promising electrochemical and antimicrobial properties, making them potential candidates for biosensor applications and antimicrobial agents.

## Data Availability

Data will be made available on request.
